# The Microbiomic Metaproteome of the Taiga Tick Ixodes persulcatus from the Tyumen Region

**DOI:** 10.32607/actanaturae.27741

**Published:** 2026

**Authors:** A. S. Kozlova, A. V. Zgoda, N. A. Petushkova, N. A. Bolochenkov, V. G. Zgoda, M. A. Salnitska, D. V. Kazakov, A. V. Lisitsa

**Affiliations:** Institute of Environmental and Agricultural Biology (X‑BIO), University of Tyumen, Tyumen, 625003 Russia; Orekhovich Institute of Biomedical Chemistry, Moscow, 119121 Russia

**Keywords:** taiga tick Ixodes persulcatus, microbiome, metaproteome, Rickettsia, Borrelia, mass spectrometry

## Abstract

Metagenomic studies have revealed the taxonomic composition of the taiga tick (Ixodes persulca tus) microbiome, whereas metaproteomic data has provided information on the biochemically active fraction of the microbial community residing in the tick. The aim of this study was to characterize the biological pro cesses taking place within the microbiome of the taiga tick I. persulcatus using a metaproteomic approach. To expand the range of identifiable proteins, we used two trypsin concentrations in sample preparation for mass spectrometric analysis. The metaproteomes of unfed female and male ticks were analyzed, which ena bled identification of protein products encoded by 2,100 genes from microorganisms belonging to 203 bacteri al and fungal species. Increased abundance of proteins associated with Ascomycota fungi, particularly abun dant in females, were detected. Proteins from the pathogenic Rickettsia and Borrelia species were identified. These findings enable a transition from a taxonomic metagenomic description to a functional analysis of the microbial consortium role in the physiology of the vector tick, particularly given the identified microbiota differences related to the tick sex.

## INTRODUCTION

Global climate change causes an expansion of the habitats of many animals, in particular arthropods, and the distribution of related pathogens throughout these newly conquered areas [1]. The fauna of ixodid ticks of the world (Acari: Ixodida) involves approximately 900 species belonging to the families Argasidae, Ixodidae, and Nuttalliellidae [2]. Currently, these ticks are found in various natural zones (from steppes to arctic deserts) and on various animals, from reptiles and birds to mammals [3].

Ixodid ticks (Ixodidae) are key reservoirs and vectors (organism vectors) of human and animal pathogens (tick-borne encephalitis, Crimean hemorrhagic fever, borreliosis, piroplasmosis, etc.) [4]. Pathogenic tick-borne viruses, bacteria, and fungi have been identified using modern sequencing methods. New pathogenic viruses have been discovered in Europe using metagenomic sequencing [5]. New bacteria of the genus *Rickettsia *have been identified by extensive studies of the tick microbiome [6, 7].

Metaproteomics complements genetic profiling (metagenomic analysis), with the characterization of metabolically active microbial taxa. Rickettsia (*Rickettsia *sp.) and Borrelia (*Borrelia *sp.) have been identified as key human pathogens transmitted by ticks inhabiting New York City parks [8]. Differences have been found between the metagenomic and metaproteomic profiles of *Ixodes *ticks in North America and Western Europe [8]. Previously, no metaproteomic studies of the taiga tick *I. persulcatus *– widespread in Russia and Asia – had been conducted.

In this study, we attempted to characterize the biological processes taking place in the taiga tick microbiome using metaproteomics.

## EXPERIMENTAL

***Ixodes persulcatus *tick samples**

Taiga *I. persulcatus *ticks were collected at the Tyumen State University Biological Station near Lake Kuchak in spring 2024 (April–May) along frequently visited nature trails. A 1 m² white flag (a thick white fabric attached to a pole) was used. Ticks caught on the flag were collected with tweezers and transferred to test tubes. After species identification based on morphological criteria, three unfed males and three unfed females were selected. The weight of the three males (15 mg) differed slightly from that of the females (13 mg). For proteomic analysis, samples were stored and transported in liquid nitrogen.

**Sample preparation for proteomic profiling**

Two pooled samples were used: sample (1) and sample (2) included three male and three female taiga ticks, respectively. Tick bodies (without limbs), frozen at –80°C, were ground with a porcelain pestle in a porcelain mortar, followed by sonication (BANDELIN Sonopuls HD 2070, Berlin, Germany) in an ice bath for two cycles of 50 s each, with a 25 s interval to reduce overheating, and solubilization in the presence of a 2% sodium dodecyl sulfate (SDS) buffer.

**Fig. 1:**
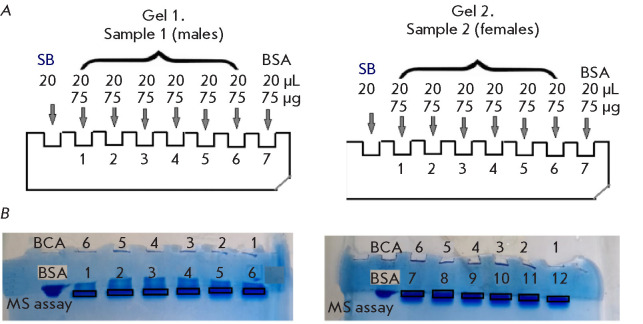
Loading of protein extracts from *Ixodes persulcatus *tick samples on 1DE gel. (*A*) SB, electrophoresis buffer, bromophenol blue. (*B*) Electropherograms of tick protein extracts. MS assay – numbering of technical replicates. BSA – bovine serum albumin, standard

The effects of SDS on the inhibition of trypsin enzymatic activity were mitigated by removing the detergent using the 1DE gel concentration procedure (SDS-PAGE without fractionating in resolving gel [9]). The scheme of sample loading on gel is presented in *Fig. 1A*. *Figure 1B* shows the resulting electropherograms.

The three most intense bands, each containing approximately 50 μg of protein, were excised from each gel. In-gel digestion was performed using the standard procedure [10]. A tryptic peptide mixture was used for mass spectrometric analysis. Mass spectra of each of the three bands excised from the gels were acquired in three technical replicates. Each of the two samples (males and females) corresponded to nine assays.

**Mass spectrometry**

Peptides were separated using high-performance liquid chromatography (HPLC, Ultimate 3000 Nano LC System, Thermo Scientific, USA) on a 15 cm C18 column with an internal diameter of 75 μm (Acclaim PepMap RSLC, Thermo Fisher Scientific). The peptides were eluted with a gradient of buffer B (80% acetonitrile, 0.1% formic acid) at a flow rate of 0.3 μL/min. The total chromatography time was 90 min. Measurements were performed on a Q Exactive HF-X Hybrid Quadrupole-Orbitrap mass spectrometer (Thermo Fisher Scientific).

**Protein identification**

Amino acid sequences of proteins characteristic of the microbial classes most abundant in the microbial communities of ixodid ticks were selected from the UniProtKB database. Four databases were compiled for Alphaproteobacteria, Gammaproteobacteria, Bacillii, and Ascomycota classes containing 36,000, 125,000, 56,000, and 35,000 verified protein-coding genes, respectively.

Peptide/protein identification was performed using the IdentiPROT software (v. 3.2) and the IdentiPy search algorithm [11]. The main identification parameters were as follows: the cleavage enzyme was trypsin; the accuracy of the match between theoretical and experimental peptide weights was ±5 parts per million, and that of fragment ions was ±0.01; the peptide ion charge state was 2+, 3+, and 4+; the number of possible missed trypsin cleavage sites was no more than one.

Sample preparation details, HPLC-MS/MS modes, and protein identification parameters are provided in the Figshare archive description at https://doi.org/10.6084/m9.figshare.29469527. The tsv files containing the metaproteome identification results are available in the [Downloads] tab at the same link.

## RESULTS AND DISCUSSION

**Identification of proteins from the taiga tick metaproteome**

The total number of unique proteins (protein groups) in microorganisms, over all classes, was 1,843, based on data for both female and male samples. If we take more than 276,000 records in UniProtKB as a normalization factor, which means the total number of known bacteria and fungi in potentially expected representatives, then metagenome coverage at the metaproteomic level is approximately 0.01% [8, 12].

On average, the number of identified microbial proteins in the *I. persulcatus *tick in sample groups did not exceed 1,100 (the number of processed mass spectra* n *= 9). For a trypsin : protein ratio of ≈ 1 : 40, sample 1 (males) contained 482 ± 97 protein identifications and sample 2 (females) contained 1,019 ± 47 protein identifications. For a trypsin : protein ratio of ≈1 : 100, sample 1 and sample 2 contained 782 ± 88 and 1,002 ± 55 protein identifications, respectively. For a trypsin : protein ratio of ≈1 : 40, the number of identified proteins in the samples of female* I. persulcatus *ticks was 2-fold higher than that in the protein extracts of male ticks. At the same time, at a trypsin : protein ratio of ≈ 1 : 100, the number of identifications in females and males did not differ significantly and the amount of bacterial and fungal proteins in SDS extracts of male ticks was only 1.3-fold lower than that in the SDS extracts of females. In samples of *I. persulcatus *males, upon addition of a smaller amount of trypsin to a gel fragment containing approximately 50–60 μg of the protein (a trypsin : protein ratio ≈1 : 100), the number of identified microbiome proteins was 1.6-fold higher than that at a trypsin : protein ratio of ≈1 : 40. In samples of *I. persulcatus *females, no similar difference in the number of identified microbiome proteins was found upon varying the trypsin : protein ratio.

Let us consider the results of protein identification of the taiga tick microbiome for nine assays representing three mass spectrometric replicates from each of three gel sections per sample. According to*Table 1*, the number of identifications is distributed depending on the sex of *I. persulcatus *ticks and the trypsin : protein ratio. The highest number of identifications was found for female samples at a lower trypsin concentration (1 : 100). Interpretation of the fragment spectra of 7,008 peptides revealed a total of 1,504 microbial proteins; i.e., 4.65 peptides per protein, on average. This indicator is in good agreement with the recommendations for assessing the quality of protein identification by mass spectrometric analysis of peptide hydrolysis products [13]. In *Table 1*, the lowest number of identifications (772) corresponds to a protein : trypsin ratio of 1 : 40 in sample 1 for the protein fraction isolated from males. Despite the lower number of identifications, the data quality is comparable to that of the assay described above (females, 1 : 100), because the peptide-to-protein ratio was 4.31.

**Table 1:** Number of microbial proteins identified using the IdentiPROT proteomic search engine based on nine mass spectra of peptide fragments (3 replicates for each of three gel sections) in a protein extract of the taiga tick I. persulcatus

Sample	Trypsin : protein ratio	Number of identifications		
		PSM^*^	peptides	proteins
No. 1, males	1 : 40	27,709	1,855	1,272
	1 : 100	37,892	2,225	1,427
No. 2, males	1 : 40	46,753	2,509	1,634
	1 : 100	48,896	2,603	1,597

^*^PSM is the Peptide Spectrum Match that is the number of experimental MS/MS mass spectra statistically significantly matching the theoretical peptide fragmentation spectrum.

**Fig. 2:**
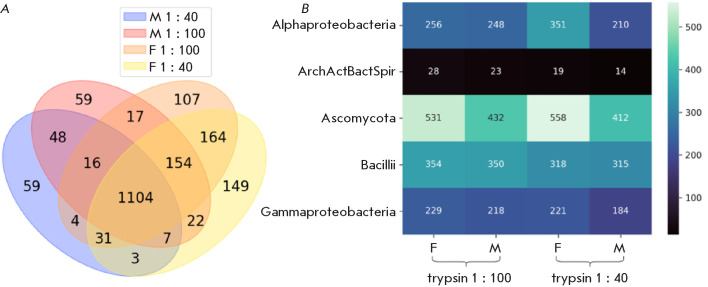
General information on the metaproteome of unfed males (M) and females (F) of the taiga tick *I. persulcatus*. Identification was performed with the IdentiProt software through databases of the main classes of bacteria and fungi using one or more peptides per protein as a criterion, with two variants of the protein : trypsin concentration ratio: 1 : 100 and 1 : 40. (*A*) The overlap of sets of identified proteins depending on the tick sex and trypsin concentration. (*B*) The heat map of the distribution of the number of identified proteins by classes of microorganisms. ArchActBactSpir* is a combination of the classes Archaea, Actinobacteria, Bacteroidia, and Spirochaeta

*Figure 2* shows diagrams of the number of identified microbial proteins from *I. persulcatus *ticks, depending on sex and the trypsin : protein ratio. The fraction of proteins common to both trypsin : protein ratio variants in the metaproteomes was 52% and 69% of the total number of identifications in males and females, respectively. It should be noted that the number of metaproteome proteins common to both males and females at the same trypsin : protein ratio was less than 50%. That is, the use of two trypsin concentrations expanded the profile of identified proteins of taiga tick microorganisms.

The heat map in *Fig. 2B* illustrates the quantitative distribution of unique proteins identified in the metaproteomes of female (F) and male (M) *I. persulcatus* ticks under different enzymatic hydrolysis conditions using trypsin at the ratios of 1 : 100 and 1 : 40. Proteins characteristic of five taxonomic classes of microorganisms (Alphaproteobacteria, ArchActBactSpir – a combined category of Archaea, Actinobacteria, Bacteroidia, and Spirochaeta, as well as Ascomycota, Bacilli, and Gammaproteobacteria) are shown. The highest number of class-specific pro-

**Microorganisms in the taiga tick metaproteome**

**Fig. 3:**
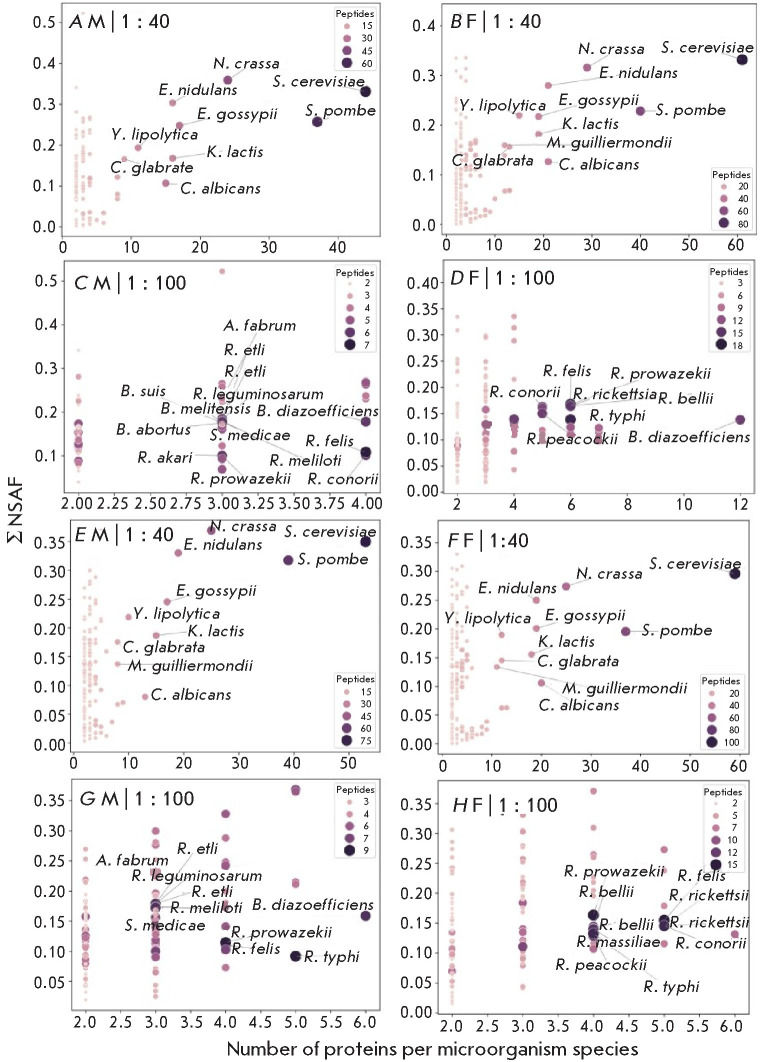
Microorganism species represented in the taiga tick metaproteome. (*A, B, E, F*) Protein : trypsin ratio = 1 : 40. (*C, D, G, H*) Protein : trypsin ratio = 1 : 100. (*A–D*) With representatives of the Ascomycete class. (*D–H*) Without the Ascomycete class. M is sample 1, males; F is sample 2, females. Σ NSAF is the sum of semiquantitative mass spectrometric estimates for proteins belonging to a particular microorganism according to annotations of the UniProtKB database

**Microorganisms in the taiga tick metaproteome** Analysis of the taiga tick metaproteome revealed the dominance of fungal proteins: ascomycetes accounted for 61% of all the identified microbial proteins (*Fig. 3A–D*). Notably, fungal proteins demonstrated exceptionally high identification reliability (more than 8 peptides per protein), whereas bacterial proteins were identified with significantly fewer peptides (less than 7; *Fig. 3E,F*). This contrast is particularly interesting in light of data [12] showing that pathogenic bacteria predominate in the metaproteome of other ixodid tick species.

We found that trypsin concentration changes did not affect the abundance of fungal proteins, which may be indication of their particular resistance to proteolysis or their high initial levels. The most abundant proteins originated from the single-celled fungi* Neurospora crassa*, *Schizosaccharomyces pombe*, and *Yarrowia lipolytica*. Notably, the semiquantitative NSAF indicator showed no significant differences between bacteria and fungi, despite the difference in the number of identified peptides.

Interestingly, the bacterial profile appears to be associated with sexual dimorphism: the bacterial spectrum in males is significantly broader and includes representatives of the *Rhizobium*, *Agrobacterium*, and* Brucella *genera (*Fig. 3*). Rickettsiae were detected in individuals of both sexes, which is consistent with data [12]. It may be suggested that the fungal component of the microbiome may play a more significant role in the physiology of the taiga tick than previously thought. The prevalence of ascomycete proteins may be considered a factor in adaptation to life on the tick’s body surface, whereas bacteria are represented primarily by endosymbionts.

**Semi-quantitative composition of the taiga tick metaproteome**

**Fig. 4:**
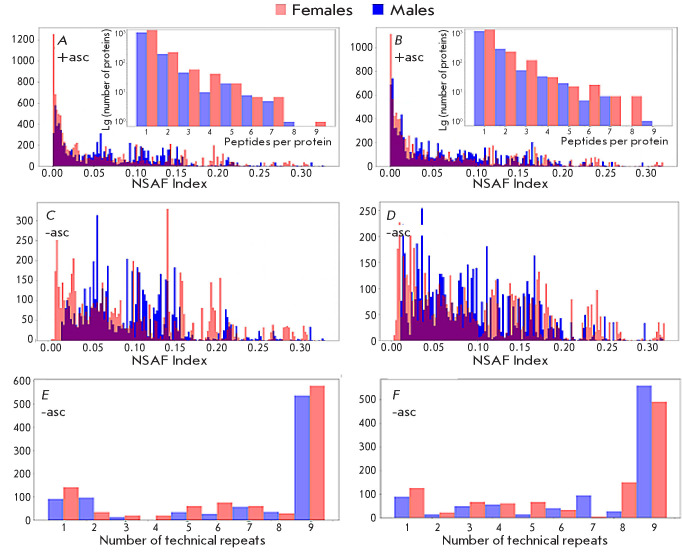
Protein composition of the taiga tick metaproteome. +/– asc denotes ascomycetes included/excluded from the analysis. Protein : trypsin ratio of 1 : 40 (*A, C*) and 1 : 100 (*B, D*). (*A, B*) Distribution of the semi-quantitative protein content estimate in samples by the NSAF index. (*C, D*) Number of the mass-spectrometry-detected peptides used to confirm protein identification. (*E, F*) “Duty cycle” means how often the identification of a protein by one or more peptides was repeated in a series of technical replicates (representatives of the Ascomycota class were excluded from analysis; designated as -asc)

*Figure 4* shows histograms of the distribution of the NSAF index, which is a relative estimate of the protein content in samples (*Fig. 4A, Fig. 4B*). Index values decrease exponentially in the range from 0.01 to the maximum protein content. The distribution became more uniform after the exclusion of proteins of the Ascomycota class: the pronounced peak seen in *Fig. 4A, Fig. 4B* in the region of low intensity values of the semi-quantitative index disappeared (*Fig. 4C, Fig. 4D*).

The insets (*Fig. 4A, Fig. 4B*) demonstrate the distribution of the number of peptides per identified protein. It should be noted that the mass spectrometric identification standards developed in the Human Proteome Project require the presence of at least two peptides [13]. However, the largest number of proteins was identified by a single peptide; these peptides unambiguously defined the sequences of the identified proteins.

The repeatability of the results in methodical replicates upon separation by one-dimensional gel electrophoresis and in technical replicates of chromatographic- mass spectrometric measurements is shown in *Fig. 4E,F* for a protein : trypsin ratio of 1 : 40 and 1 : 100, respectively. It is evident that approximately 500 proteins were identified in both males and females in all nine experimental replicates. The estimated number of identifications in male and female ticks at different amounts of the hydrolytic agent is >1,800 protein products. About a quarter of these are characterized by high identification repeatability in an experiment involving 9 × 2 = 18 technical replicates. Information on these reliably identified proteins is shown in *Table 2*.

**Table 2:** Highly abundant bacterial proteins in the metaproteomes of male and female taiga ticks^*^

No.	Protein	Microorganism	Average NSAF^**^±SD		Mol. weight, kDa
			Females	Males	
1.	ATP synthase, alpha subunit	Saccharophagus degradans, Teredinibacter turnerae, Dichelobacter nodosus, Francisella tularensis, Pseudomonas putida, etc.	0.22 ± 0.04	0.18 ± 0.05	~55
2.	ATP synthase, beta subunit	Phocaeicola vulgatus, Bacillus cytotoxicus, B. cereus, B. thuringiensis, B. anthracis, etc.	0.21 ± 0.06	0.24 ± 0.04	~52
3.	ATP synthase, beta subunit	Glaesserella parasuis	n/a	0.21 ± 0.05	~55
4.	Chaperone DnaK	Thermosipho africanus, Bartonella henselae, Bartonella tribocorum, Azorhizobium caulinodans	0.18 ± 0.03	0.15 ± 0.05	~65
5.	Chaperone HtpG	Buchnera aphidicola, Rickettsia felis, R. bellii, Rickettsia typhi	0.12 ± 0.04	0.12 ± 0.08	51–72
6.	NADP-dependent isocitrate dehydrogenase	Sphingobium yanoikuyae	0.07 ± 0.01	0.04 ± 0.01	~45
7.	Chaperone GroEL	R. rickettsii, R. prowazekii, R. bellii, R. typhi	n/a	0.08 ± 0.02	~52

^*^A total protein content to a hydrolytic enzyme (trypsin) ratio of 1 : 100 (millimolar concentrations).

^**^NSAF is an index showing the amount of protein analyte determined by summing mass spectrometric signals in the absence of an isotope-labeled peptide standard (Normalized Spectral Abundance Factor). n/a – no data, SD – deviation from the mean NSAF index value.

*Table 2* presents the proteins identified in nine technical replicates (three HPLC-MS runs for each of the three gel fragments) in males and females. Metaproteome analysis identified seven housekeeping proteins in each of the nine instrument runs. The core was constituted by energy metabolism enzymes (ATP synthase, malate dehydrogenase, isocitrate dehydrogenase) and stress-inducible chaperones (DnaK, HtpG, GroEL).

Interestingly, the synthesis levels of malate dehydrogenase and chaperone GroEL were persistently higher in females. Of particular note was the role of ATP synthase found in 39 bacterial species; ATP synthase subunits accounted for over 60% of the reproducible mass spectrometric identifications. A semi-quantitative assessment (NSAF) confirmed the abundance of ATP synthase (NSAF > 0.20), with minimal values for the chaperone GroEL (NSAF = 0.08). The increased abundance of chaperones in females may reflect an adaptive strategy of the microbiome to maintain protein homeostasis and ensure the survival of both commensal and pathogenic microorganisms under physiological stress [14]. The ability to maintain viability during host switching, that is, during the transition from a tick to a warm-blooded host, is particularly important for pathogens such as *Borrelia* spp.

**Fig. 5:**
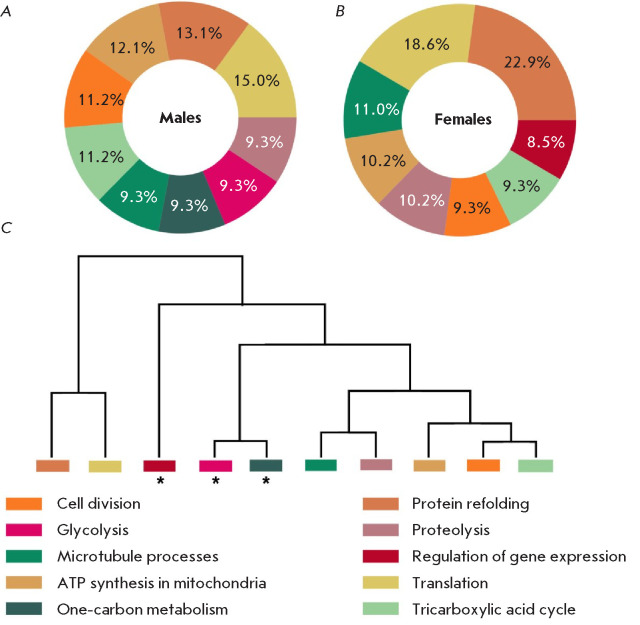
Protein distribution in the microbiome metaproteome of unfed male (*A*) and female (*B*) *Ixodes persulcatus* ticks by biological process categories (Gene Ontology : Biological Process). (*C*) Clustering of metabolic processes. The * symbol denotes protein groups whose content differs significantly in the microbiome metaproteomes of males and females

*Figure 5* presents the results of the functional classification of the proteins in the microbiome metaproteome of male and female *I. persulcatus *ticks. Doughnut charts reflect the relative abundance of proteins involved in biological processes (Gene Ontology : Biological Process, GO : BP) in the metaproteome profile (*Fig. 5A*, males; *Fig. 5B*, females). The analysis revealed significant sex differences in the functional profile of the *I. persulcatus *microbiome. In males, the processes of cell division (15.0%) and ATP synthesis (12.1%) dominate, which indicates the orientation of the microbiota towards maintaining energy-dependent functions and proliferative activity. In females, proteins associated with refolding (22.9%) and cell division (18.6%) predominate, which is related to enhanced protein quality control and proliferative activity. Our data are consistent with the results of [12] emphasizing an increased level of biosynthesis of housekeeping proteins in the metaproteome of the female ticks of another species, *I. ricinus*.

The dendrogram (*Fig. 5C*) reveals functional similarities between biological processes. The dendrogram structure includes clusters combining energy metabolism and regulatory mechanisms (glycolysis, onecarbon metabolism, regulation of gene expression), as well as processes maintaining protein homeostasis (protein refolding and translation). An individual cluster summarizes catabolic pathways and cell division, which are coupled with the tricarboxylic acid cycle, apparently reflecting the coordination of energy metabolism and proliferative processes.

## CONCLUSIONS

We studied the metaproteome of I. persulcatus, a vector of tick-borne encephalitis and borreliosis in Eurasia, using a metaproteomic approach. Analysis of metaproteomes of the taiga tick I. persulcatus re quired an optimization of the sample preparation pro cedure, which resulted in a 19% increase in the num ber of identified proteins using a combined detergent removal approach. We functionally characterized the proteomic composition of the tick microbiome. We moved from taxonomic lists, known from metagenom ic data, to the assessment of the biochemical activity of the microbial consortium. In the metaproteome, we found elevated contents of proteins associated with Ascomycota fungi, which was particularly pronounced in females. The identified metaproteome profile, dom inated by fungal proteins, differs from data previous ly reported for other tick species (e.g., I. scapularis [8]). Proteins from pathogenic bacteria, rickettsia and borrelia, were observed in the metaproteome. A total of 2,100 microbe-specific proteins were identified, indicating the high diversity of the blood-sucking tick microbiota.

It should be considered that low microbial pro teome coverage in the setting of a dominant pool of tick host proteins is characteristic of the survey meta proteomics approach for complex samples. This inevi tably leads to an underestimation of the fraction of low-abundant, but possibly critically important, patho gens. Their detection requires pre-separation of mi crobial cells using microfluidics, in combination with targeted proteomic approaches [15].

Therefore, we have been able to identify biochemi cal processes in the microbiota of the taiga tick I. per sulcatus (*Fig. 5*). The obtained data include both bac terial and fungal gene products. The obtained data on the tick microbiota metaproteome are important for developing a multi-omics understanding of I. persul catus activity.

## References

[1] Montero E., González LM., Chaparro A. (2016). First record of Babesia sp. in Antarctic penguins.. Ticks Tick Borne Dis..

[2] Horak IG., Camicas JL., Keirans JE. (2002). The Argasidae, Ixodidae and Nuttalliellidae (Acari: Ixodida): A World List of Valid Tick Names.. Exp Appl Acarol..

[3] Beati L., Klompen H. (2019). Phylogeography of Ticks (Acari: Ixodida).. Annu Rev Entomol..

[4] Tsapko NV. (2020). A Checklist of the ticks (Acari: Ixodidae) of Russia.. Parazitologiya..

[5] Ergunay K., Golubiani G., Kirkitadze G. (2025). Ongoing circulation of emerging tick-borne viruses in Poland, Eastern Europe.. PLoS One..

[6] Polsomboon Nelson S., Ergunay K., Bourke BP. (2024). Nanopore-based metagenomics reveal a new Rickettsia in Europe.. Ticks Tick Borne Dis..

[7] Ergunay K., Boldbaatar B., Bourke BP. (2024). Metagenomic Nanopore Sequencing of Tickborne Pathogens, Mongolia.. Emerg Infect Dis..

[8] Smith HR., Canessa EH., Roy R., Spathis R., Pour MS., Hathout Y. (2022). A single tick screening for infectious pathogens using targeted mass spectrometry.. Anal Bioanal Chem..

[9] Shkrigunov T., Pogodin P., Zgoda V. (2022). Protocol for Increasing the Sensitivity of MS-Based Protein Detection in Human Chorionic Villi.. Curr Issues Mol Biol..

[10] Shevchenko A., Wilm M., Vorm O., Mann M. (1996). Mass Spectrometric Sequencing of Proteins from Silver-Stained Polyacrylamide Gels.. Anal Chem..

[11] Levitsky LI., Ivanov MV., Lobas AA. (2018). IdentiPy: An Extensible Search Engine for Protein Identification in Shotgun Proteomics.. J Proteome Res..

[12] Kamburov A., Cavill R., Ebbels TM., Herwig R., Keun HC. (2011). Integrated pathway-level analysis of transcriptomics and metabolomics data with IMPaLA.. Bioinformatics..

[13] Paik YK., Omenn GS., Uhlen M. (2012). Standard Guidelines for the Chromosome-Centric Human Proteome Project.. J Proteome Res..

[14] Henderson B., Allan E., Coates AR. (2006). Stress Wars: the Direct Role of Host and Bacterial Molecular Chaperones in Bacterial Infection.. Infect Immun..

[15] Terekhov SS., Smirnov IV., Malakhova MV. (2018). Ultrahigh-throughput functional profiling of microbiota communities.. Proc Natl Acad Sci U S A..

